# Assessment of hepatitis C virus permissiveness in iteratively genetically humanized mice

**DOI:** 10.1128/jvi.00793-25

**Published:** 2025-09-03

**Authors:** Michael P. Schwoerer, Sebastian Carver, Thomas R. Cafiero, Bradley Joyce, Keith A. Berggren, Saori Suzuki, Brigitte Heller, Aoife K. O'Connell, Hans P. Gertje, Nicholas A. Crossland, Eszter Posfai, Alexander Ploss

**Affiliations:** 1 Department of Molecular Biology, Princeton University 6740, Princeton, New Jersey, USA; 2 National Emerging Infectious Diseases Laboratories, Boston University 1846, Boston, Massachusetts, USA; 3 Department of Pathology and Laboratory Medicine, Boston University Chobanian & Avedisian School of Medicine, Boston, Massachusetts, USA; Wake Forest University School of Medicine, Winston-Salem, North Carolina, USA

**Keywords:** hepatitis C virus, HCV, host tropism, innate immunity, animal models

## Abstract

Hepatitis C virus (HCV) is an enveloped, positive-sense single-stranded RNA virus causing chronic infections in over 50 million people who are at risk of developing severe liver disease. Greater understanding of HCV pathogenesis and vaccine development has been hampered by the lack of a fully immunocompetent small-animal model permissive to infection. Rodents are resistant to HCV infection due to a variety of factors at the levels of entry and replication, many of which have been discovered within the past decade. We hypothesized that genetically altering these factors in mice would provide a host environment conducive to infection. Here, we present the generation and characterization of a series of mouse lines bearing humanized alleles for CD81, occludin, TRIM26, and CypA, the murine orthologs for which do not support HCV uptake and replication. Additionally, we knocked out CD302 and CR1L, which restrict HCV infection in mouse hepatocytes. Intravenously, inoculation of mice harboring some or all of these mutant alleles did not increase viremia. To ascertain that mouse adaptive immune responses do not rapidly clear any putative low-level viremia, we engrafted hepatocytes from these genetically complex lines into immunodeficient liver-injury strains. No cohort of mice presented with sustained HC viremia, although we detected low-level viremia in a subset of transplant-recipient mice. Collectively, although these mouse models did not support robust, sustained viremia, these mouse mutant lines represent the most genetically advanced mouse model of HCV infection and will provide an important platform for future genetic host adaptations and/or complementary viral adaptation approaches.

**IMPORTANCE:**

Hepatitis C virus (HCV) presents a significant threat to global health. Despite its prevalence worldwide, there remain significant knowledge gaps regarding immunopathogenesis, oncogenesis, and determinants for vaccine efficacy. This is due to the scarcity of small-animal models for HCV, a virus that only robustly infects human and chimpanzee hepatocytes. In this work, we genetically engineer mice to either humanize or remove several factors that are known to limit HCV infection in mice. We then expose these mice to HCV and assess whether they develop infection over time. To see whether the immune system impacts infection in these modified mice, we transplant liver cells from those mice into ones that lack immune cells and then assess their ability to develop HCV infection. While we did not succeed in generating a mouse that sustains robust viremia, these complex strains nevertheless represent an important platform for further model development.

## INTRODUCTION

Hepatitis C virus (HCV) is the etiological agent of hepatitis C, a liver disease afflicting more than 58 million people globally (1, 2). HCV is an enveloped, positive-sense single-stranded RNA hepacivirus within the family *Flaviviridae* (3). Despite the efficacy of direct-acting antivirals (DAAs) to resolve HCV infection, a vaccine that precludes either initial infection or the development of chronicity has yet to be generated (4). This is consequential, as untreated HCV infection often progresses to a chronic disease characterized by extensive inflammation and bridging fibrosis within the liver (5). This, in turn, results in decompensated cirrhosis and ultimately hepatocellular carcinoma or end-stage liver disease. Notably, despite the aforementioned DAAs, more individuals are (re-)infected than cured annually, highlighting the need for a prophylactic HCV vaccine (4).

Animal models are vital to vaccine development, enabling rigorous testing of vaccine candidates and prioritization for clinical trials. The lack of an immunocompetent model for HCV infection presents yet another hurdle for vaccine developers to clear, in addition to the antigenic diversity of HCV across/within genotypes. Beyond vaccine development, a mouse model for HCV infection would facilitate studies of HCV pathogenesis. HCV has a remarkably narrow tropism; robust, replicable infection occurs solely in the hepatocytes of humans and chimpanzees. While chimpanzees were undoubtedly a critical model for HCV research (6), the moratorium on their use in biomedical research has created a pressing need for alternative models.

To address this need for a small-animal model for HCV infection, numerous orthogonal approaches have been taken (7). For example, immunodeficient liver-injury strains, such as the constitutive urokinase-type plasminogen activator transgenic (8, 9) and fumarylacetoacetate hydrolase (FAH)-deficient models (10, 11), engrafted with human hepatocytes have been a powerful tool for understanding the dynamics of HCV infection *in vivo*, though these systems lack the immune cell types implicated in long-term liver pathology (12). Dual engraftment of human liver-chimeric mice with human hematopoietic stem cells can provide further insights into immune-mediated mechanisms of liver injury and disease pathogenesis, albeit without a human-like B cell activation that would be useful for vaccine development (13). Surrogate hepaciviruses that cause HCV-like disease in animals, including equine hepacivirus in horses (14), GBV-B in New World monkeys (15), and Norway rat hepacivirus (NrHV) in rats (16, 17) and mice (16, 18), have been leveraged extensively to study pathogenesis (16) and determinants for adaptive immune protection (19). The utility of such models is inherently limited due to the sequence and antigenic dissimilarity of these viruses to HCV (20–22). There have additionally been efforts to establish the treeshrew *Tupaia belangeri* as a model system for HCV infection, given reports of viremia in a subset of infected animals (23). However, this model lacks existing resources for genetic manipulation, and researchers have remarked on the difficulty of breeding and maintaining colonies of *Tupaia*. Lastly, mice have been generated that stably express the entire HCV polyprotein or individual proteins, an approach that has been beneficial for hepatitis B virus research *in vivo* (reviewed in reference 24). Whereas these have been useful models for understanding HCV-driven liver pathologies, these mice do not develop viremia.

The preferred small-animal model for HCV infection would be an immunocompetent mouse, due to their widespread use in biomedical research and relatively economical husbandry. This has been achieved for a variety of other RNA viruses, including poliovirus (25), Zika virus (ZIKV) (26), severe acute respiratory syndrome coronavirus 1 (SARS-CoV-1) (27), and SARS-CoV-2 (27, 28); however, these species-promiscuous viruses solely required the humanization of a single factor. Given the narrow tropism of HCV, it can be presumed that a more complex genetic background would be required to permit infection. *In vitro* data suggest that such a model may be generated, to the extent that murine hepatocytes can support low-level replication of drug-selectable HCV replicons.

Multiple barriers to HCV infection exist in mice, as has been reviewed previously (29). Murine cluster of differentiation 81 (CD81) and occludin (OCLN) are unable to facilitate HCV entry, whereby expression of the human orthologs permits entry in concert with murine scavenger receptor class B type 1 (SCARB1) and claudin 1 (CLDN1) (30). Murine CD302 and complement C3b/C4b receptor 1 like (CR1L) are surface-bound restriction factors that inhibit cell entry by cooperatively binding HCV E1E2 heterodimers (31). Whereas human CD302 can still inhibit HCV entry, human CR1L is unable to do so, limiting the reported cooperative antiviral activity of this system. The murine ortholog of tripartite motif 26 (TRIM26), an E3 ubiquitin ligase necessary for the formation of the HCV replication organelle (RO) in human infections, is unable to interact with HCV NS5B, accordingly abrogating ubiquitination and RO establishment (32). While human cyclophilin A (CypA) is a well-known host factor involved in the HCV reproductive cycle (33, 34), the murine ortholog has additionally been demonstrated to be incapable of supporting HCV replication (35).

Given the improvements to HCV infection observed in genetically humanized mouse cells *in vitro*, along with incremental improvements to replication in immune-suppressed strains, we sought to derive a mouse line harboring modifications in all factors robustly demonstrated to be important to HCV species specificity. To this end, we conducted an iterative process of genetic engineering in mice, targeting loci encoding CD81, OCLN, CD302, CR1L, TRIM26, and CypA. We assayed the permissiveness of intermediate lines to HCV infection in immunocompetent and immunodeficient backgrounds.

## RESULTS

### Generation of CD81^EL2[H/H]^ OCLN^EL2[H/H]^ (EFKI) C3^-/-^ CR1L^-/-^ CD302^-/-^ (3C) mice

**Fig 1:**
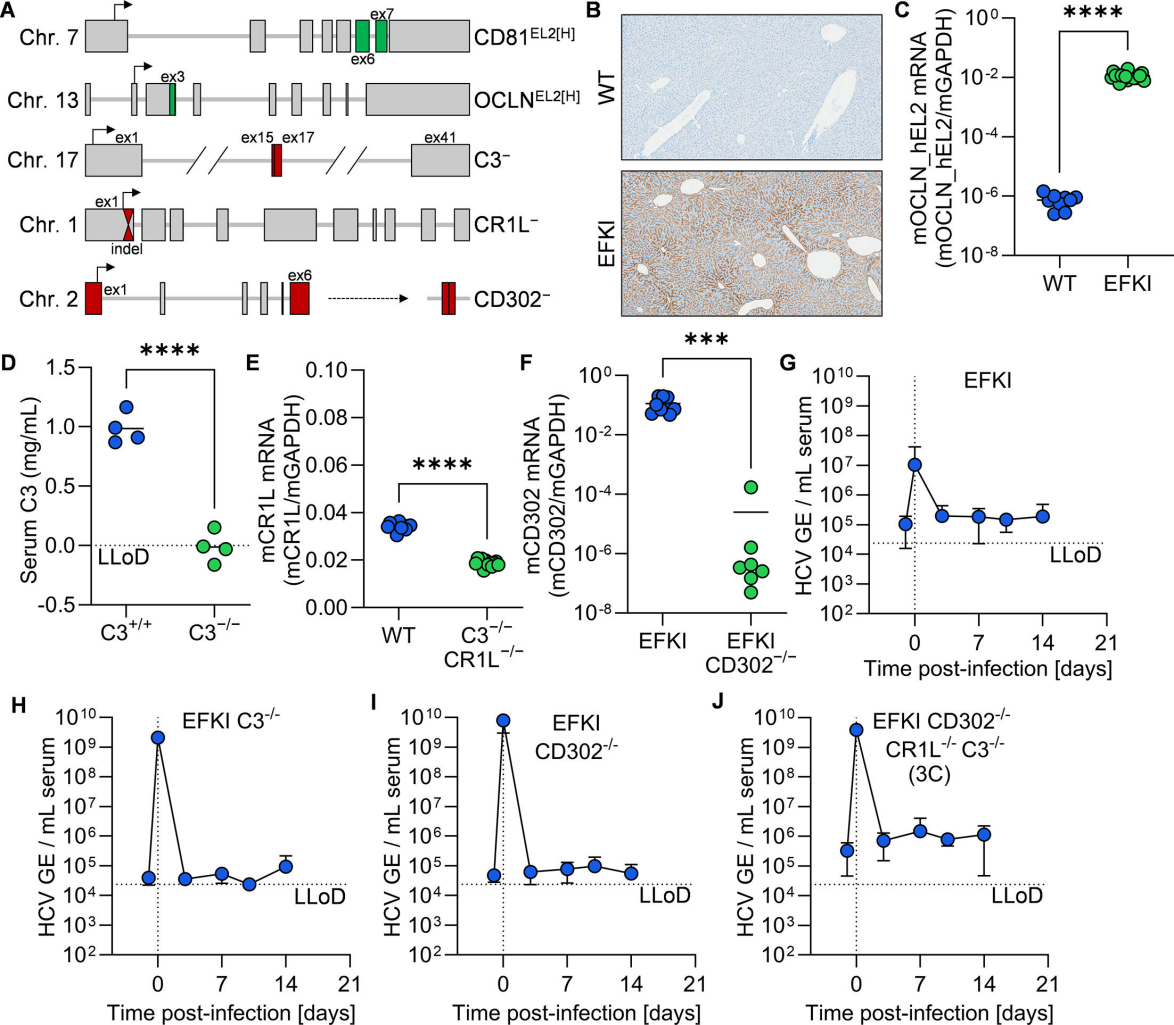
HCV-susceptible mice deficient in CD302, CR1L, and C3 are not permissive to HCV infection. (**A**) Schematic representation of modified loci utilized (CD81, OCLN, C3) or newly generated (CD302, CR1L) for this study. (**B**) CD81 staining (brown) of representative liver sections from wild-type (WT) and CD81^EL2[H/H]^ OCLN^EL2[H/H]^ (EFKI) mice (400× magnification). (**C**) Quantification of human OCLN EL2-specific transcript in liver tissue from WT and EFKI mice. ****, *P* ≤ 0.0001. (**D**) Quantification of serum C3 concentration in C3^+/+^ and C3^-/-^ mice. ****, *P* ≤ 0.0001. (**E**) Quantification of murine CR1L-specific transcript in liver tissue from WT and CR1L^-/-^ C3^-/-^ mice. ****, *P* ≤ 0.0001. (**F**) Quantification of murine CD302-specific transcript in liver tissue from EFKI and EFKI CD302^-/-^ mice. ***, *P* ≤ 0.001. (**G–J**) Longitudinal HCV serum viremia in (**G**) EFKI (*N* = 5), (**H**) EFKI C3^-/-^ (*N* = 5), (**I**) EFKI CD302^-/-^ (*N* = 5), and (**J**) EFKI CD302^-/-^ CR1L^-/-^ C3^-/-^ (3C) (*N* = 6) mice. LLoD, lower limit of detection.

To create an HCV mouse model, we began with a previously generated mouse line that harbors minimally humanized HCV entry factors (36). These entry-factor knock-in (EFKI) mice contain alleles for murine CD81 and OCLN in which the second extracellular loop (EL2) of the native locus has been replaced with the orthologous human sequence (Fig. 1A) (36). We confirmed expression of the CD81^EL2[H]^ and OCLN^EL2[H]^ alleles by means of histology staining (Fig. 1B) and, due to lack of an anti-OCLN antibody suitable for distinguishing the murine and humanized OCLN protein, RT-qPCR (Fig. 1C).

We next sought to knock out the murine cell-surface restriction factors CD302 and CR1L. CR1L knockout mice are subject to embryonic lethality in a complement-mediated mechanism (37), necessitating any deletion to be conducted on a C3-deficient strain in which all three major complement effector functions are compromised (38). We utilized an extant C3 knockout line bearing a large deletion spanning coding exons 15 and 17 (Fig. 1A). We confirmed C3 deficiency in this line by means of a commercial enzyme-linked immunosorbent assay (ELISA) kit (Fig. 1D) and bred EFKI C3^-/-^ mice.

Next, we knocked out CR1L in EFKI C3^-/-^ utilizing an approach similar to two-cell homologous recombination CRISPR (2C-HR-CRISPR) (39). However, instead of introducing a repair template for homologous recombination, we used two guide RNAs flanking a critical exon to generate double-strand breaks. This resulted in a seven-nucleotide deletion encompassing nucleotide positions +58 to +64 that corresponds to a 24 amino acid product (Fig. 1A). The first 40 codons of the CR1L locus encode the signal peptide, rendering this product non-functional (31). Consistent with a mechanism of mRNA nonstop decay, we detected significantly lower CR1L RNA levels in C3^-/-^ CR1L^-/-^ liver tissue via RT-qPCR (Fig. 1E).

Separately, we knocked out CD302 in EFKI and EFKI C3^-/-^ CR1L^-/-^ mice, utilizing gRNAs targeting coding exons 1 and 6 (Fig. 1A). Again, due to the lack of antibodies suitable for detecting CR1L or CD302, we confirmed knockdown by RT-qPCR on liver tissue, finding a significant decrease in CD302 transcript levels in the EFKI CD302^-/-^ line (Fig. 1F).

### EFKI, EFKI C3^-/-^, EFKI CD302^-/-^, and EFKI 3C mice are not permissive to HCV infection

Having generated an HCV-susceptible mouse line lacking CD302 and CR1L, we aimed to assess the permissiveness of the resultant EFKI CD302^-/-^ CR1L^-/-^ C3^-/-^ (3C) mice, along with several intermediate strains. We injected mice intravenously with 1E6 tissue culture infectious doses (TCID) of the genotype 2a/2a intergenotypic HCV J6/JFH1-Jc1 strain, which was previously shown to be infectious in humanized mice (40). To affirm that the mice received a proper inoculum, we bled mice immediately after infection. We proceeded to bleed mice longitudinally at days 3, 7, 10, and 14 post-infection. For EFKI (Fig. 1G), EFKI C3^-/-^ (Fig. 1H), EFKI CD302^-/-^ (Fig. 1I), and EFKI 3C (Fig. 1J) mice, we did not observe any increases in detectable HCV RNA copy numbers in serum above pre-exposure levels. This is consistent with what has been observed previously in mice in which human OCLN and CD81 (hOC) are overexpressed on a CD302-deficient background; these hOC CD302^-/-^ mice are similarly refractory to infection with HCV J6/JFH1-Jc1 (31).

### Generation of FNRG[EFKI 3C] mice

**Fig 2:**
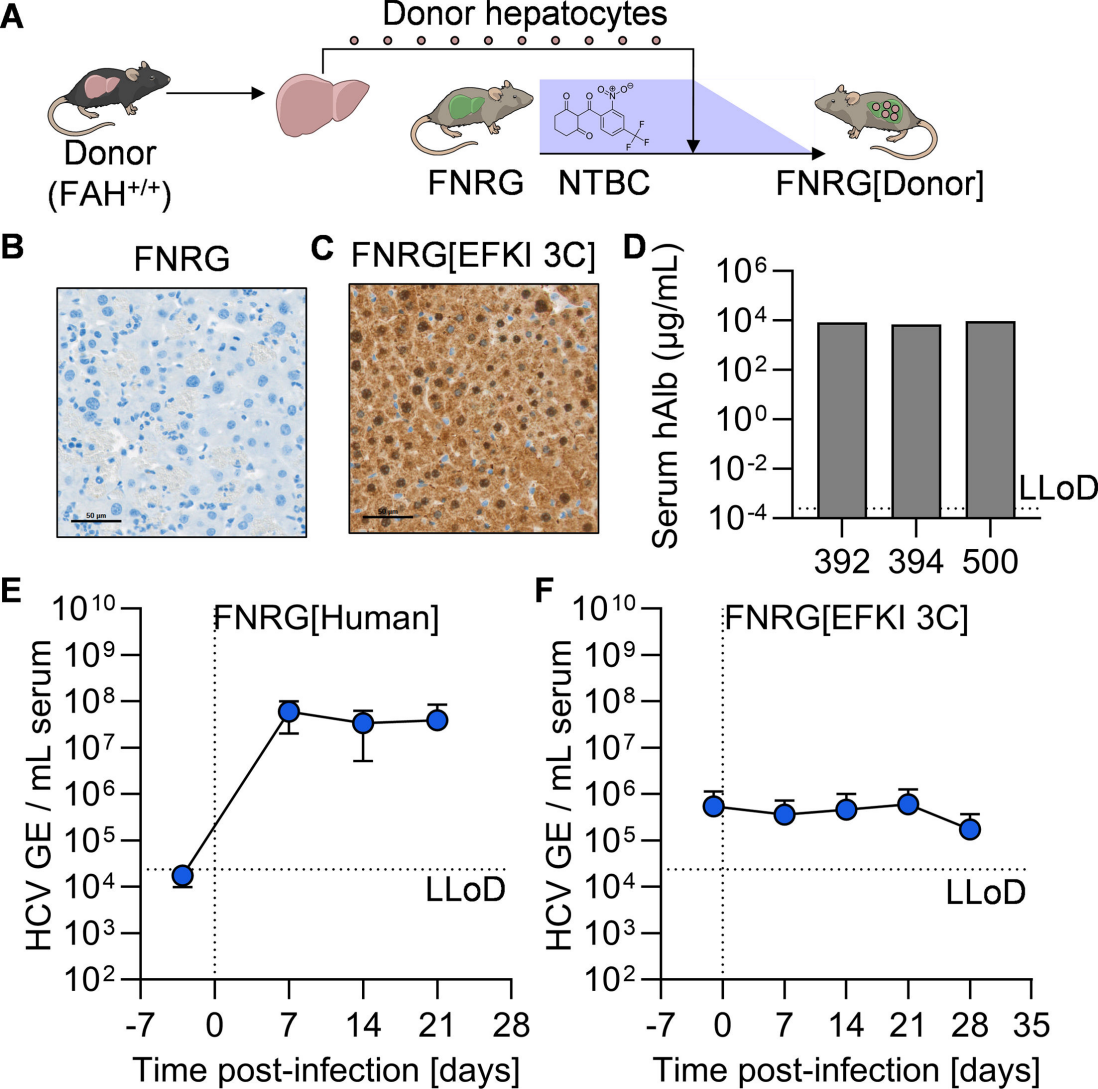
Immunodeficient mice harboring EFKI C3^-/-^ Cr1L^-/-^ CD302^-/-^ (3C) hepatocytes are not permissive to HCV infection. (**A**) Schematic representation of the experimental design of engrafting (FAH^+^) hepatocytes isolated from EFKI 3C mice into FNRG recipients. FAH staining (brown) of liver sections from representative samples from (**B**) FNRG and (**C**) FNRG[EFKI 3C] mice. Scale bar = 50 µm. (**D**) Quantification of serum human albumin concentration in FNRG[Human] mice. (**E–F**) Longitudinal HCV serum viremia in (**E**) FNRG[Human] (*N* = 3) and (**F**) FNRG[EFKI 3C] (*N* = 14) mice. LLoD, lower limit of detection.

We hypothesized that the apparent lack of viremia in EFKI 3C mice could derive from the activity of murine antiviral immunity. To this end, we generated a cohort of immunodeficient, transplant-recipient mice engrafted with EFKI 3C hepatocytes. FAH^-/-^ non-obese diabetic (NOD) recombinase activating gene 1 (Rag1^-/-^) interleukin 2 receptor gamma deficient (Il2rg^NULL^, FNRG) mice are an established platform for programmable liver injury and hepatocyte engraftment (11). Functional B, T, and NK cells are absent in FNRG mice, and lifelong provision of the drug 2-(2-nitro-4-trifluoromethylbenzoyl)-1,3-cyclohexanedione (NTBC) is necessary to prevent hepatotoxicity (11). After intrasplenic injection of FNRG mice with FAH^+^ donor hepatocytes, NTBC is withdrawn, enabling selective depletion of the endogenous FAH^-/-^ hepatocytes and stimulating proliferation of donor hepatocytes in the murine parenchyma (Fig. 2A).

### FNRG[EFKI 3C] mice are not permissive to HCV infection

At 7 weeks post-injection of EFKI 3C hepatocytes into FNRG mice, we assessed engraftment in the resultant FNRG[EFKI 3C] mice. Staining liver samples for the presence of FAH, we detected no signal in the original FNRG mice (Fig. 2B) and strong signal in EFKI 3C hepatocyte-injected mice (Fig. 2C), indicating high engraftment in our FNRG[EFKI 3C] cohort. We separately generated a cohort of mice transplanted with primary human hepatocytes (FNRG[Human]), the engraftment of which we confirmed utilizing an ELISA for human serum albumin (Fig. 2D). We injected these mice intravenously with 1E6 TCID HCV J6/JFH1-Jc1 and monitored serum HCV RNA longitudinally; post-bleeds immediately following inoculation were omitted in this and all subsequent FNRG cohorts due to the fragility of the model. In line with previous studies, injecting a human liver chimeric mouse with the same inoculum resulted in robust viremia which was stably maintained for several weeks (Fig. 2E) (9–11). As with the immunocompetent EFKI 3C mice, we observed no increase in viremia in FNRG[EFKI 3C] mice (Fig. 2F).

### Humanization of the murine TRIM26 locus fails to convey permissiveness in EFKI, EFKI CD302^-/-^, or EFKI 3C mice

**Fig 3:**
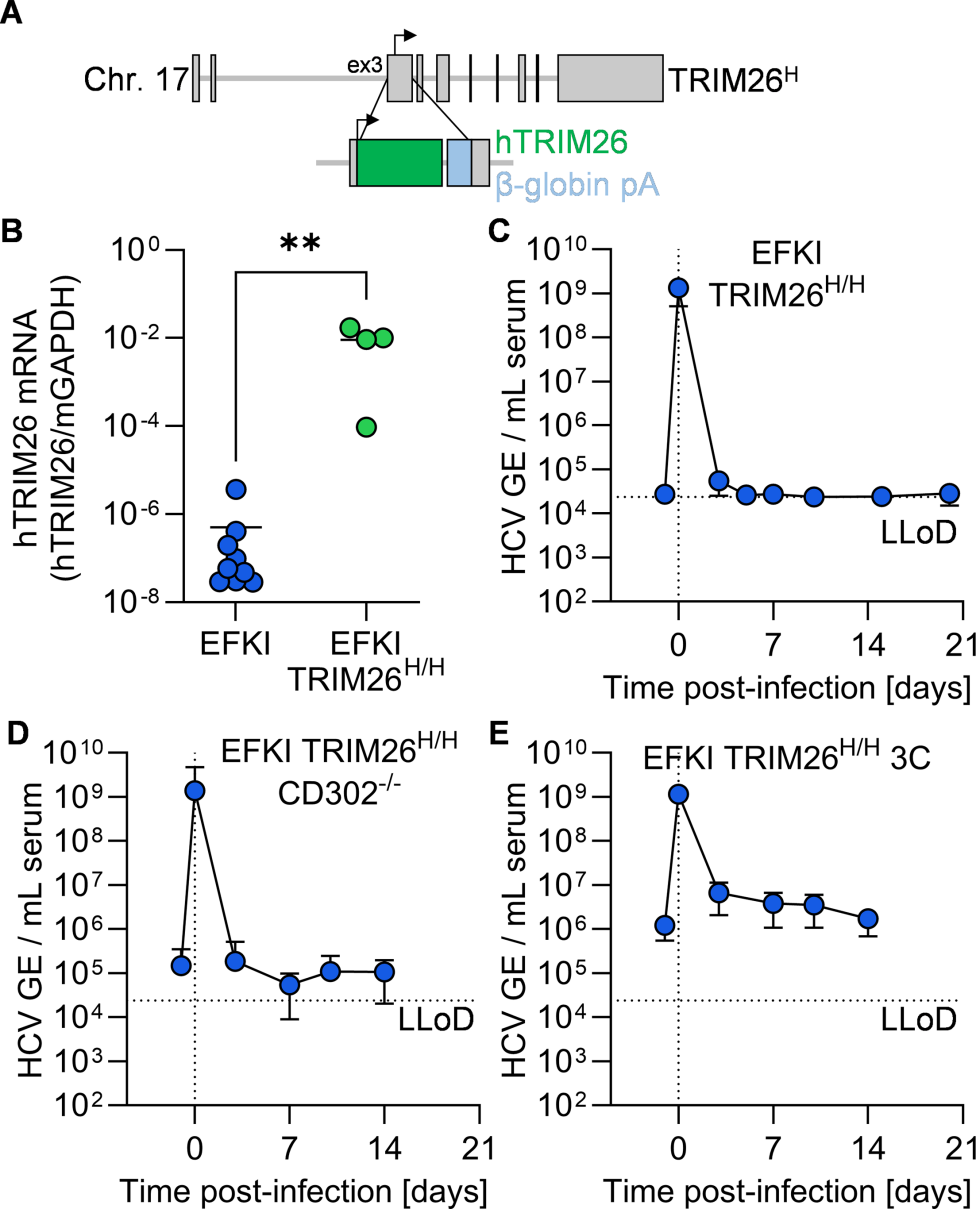
Humanization of TRIM26 does not convey HCV permissiveness *in vivo*. (**A**) Schematic representation of the humanized TRIM26 locus generated for this study. (**B**) Quantification of human TRIM26-specific transcript in liver tissue from EFKI and EFKI TRIM26^H/H^ mice. **, *P* ≤ 0.01. (**C–E**) Longitudinal HCV serum viremia in (**C**) EFKI TRIM26^H/H^ (*N* = 6), (**D**) EFKI TRIM26^H/H^ CD302^-/-^ (*N* = 9), and (**E**) EFKI TRIM26^H/H^ 3C (*N* = 8) mice. LLoD, lower limit of detection.

We separately humanized the murine TRIM26 locus, a factor that had previously been demonstrated to be necessary for forming HCV ROs during infection. We knocked out the native murine locus while simultaneously knocking in the coding sequence for human TRIM26. To do this, we conducted 2C-HR-CRISPR, utilizing gRNAs targeting the region immediately preceding the start codon in exon 3 of the murine TRIM26 locus, along with a repair template encoding full-length human TRIM26 and a rabbit β-globin polyA sequence (Fig. 3A). This allows the human TRIM26 knock-in to be expressed in the same 5′ regulatory regime as the murine ortholog, while at the same time disrupting expression of murine TRIM26. We confirmed the expression of human TRIM26 in EFKI TRIM26^H/H^ mouse liver by RT-qPCR due to the lack of human TRIM26-specific antibodies, showing a significant increase in mRNA expression compared to EFKI (Fig. 3B). We additionally incorporated the knock-in TRIM26^H^ allele into EFKI CD302^-/-^ and EFKI 3C mice.

We assessed HCV permissiveness in human TRIM26 knock-in mice in the same fashion as in Fig. 1G through J. Again, we did not detect increased viremia above background levels in EFKI TRIM26^H/H^ (Fig. 3C), EFKI CD302^-/-^ TRIM26^H/H^ (Fig. 3D), and EFKI 3C TRIM26^H/H^ mice (Fig. 3E).

### Variable HCV permissiveness of FNRG[EFKI TRIM26^H/H^ CD302^-/-^] and FNRG[EFKI TRIM26^H/H^ 3C] mice

**Fig 4:**
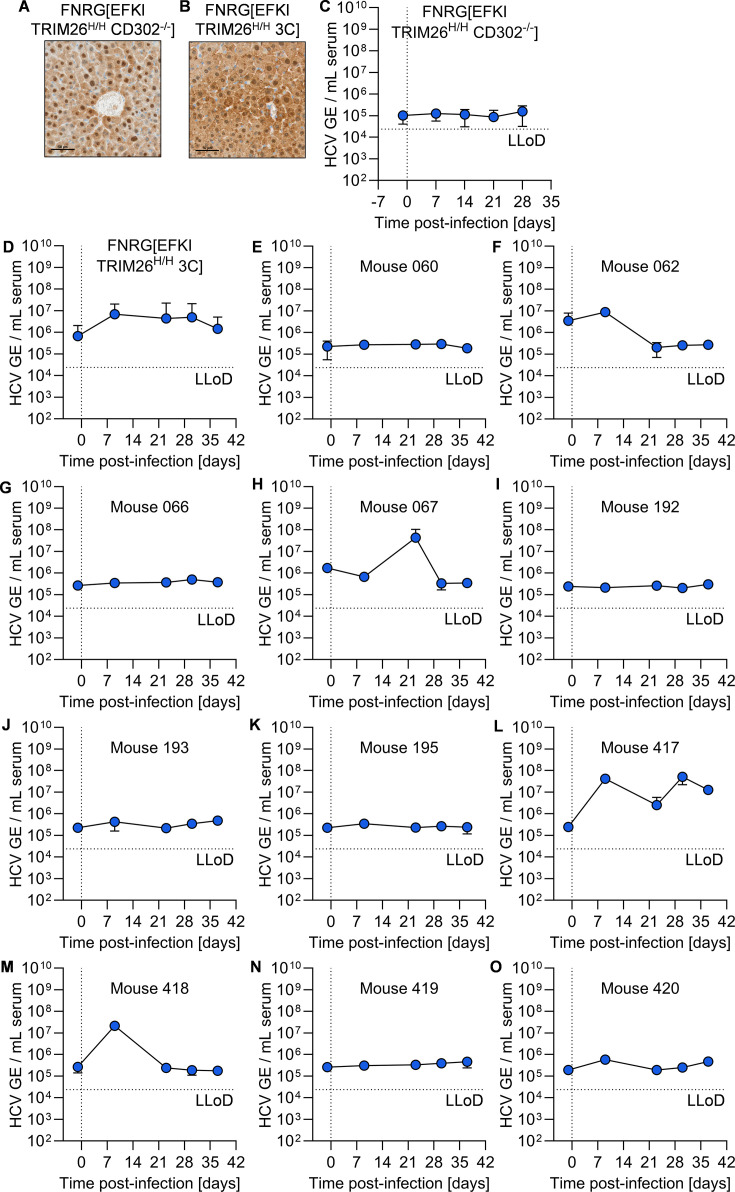
Immunodeficient mice harboring EFKI TRIM26^H/H^ CD302^-/-^ or EFKI TRIM26^H/H^ 3C hepatocytes are not permissive to HCV infection. (**A and B**) FAH staining (brown) of liver section from representative liver samples from (**A**) FNRG[EFKI TRIM26^H/H^ CD302^-/-^] and (**B**) FNRG[EFKI TRIM26^H/H^ 3C] mice. Scale bar = 50 µm. (**C and D**) Longitudinal HCV serum viremia in (**C**) FNRG[EFKI TRIM26^H/H^ CD302^-/-^] (*N* = 8) and (**D**) FNRG[EFKI TRIM26^H/H^ 3C] (*N* = 11) mice. LLoD, lower limit of detection. (**E–O**). Longitudinal serum viremia of individual mice in (**D**).

We next generated cohorts of FNRG transplant-recipient mice engrafted with EFKI TRIM26^H/H^ and EFKI TRIM26^H/H^ 3C hepatocytes, both of which showed robust engraftment, as assessed by FAH histology staining (Fig. 4A and B). Following injection of HCV, we did not see any longitudinal increase in viremia above baseline in FNRG[EFKI TRIM26^H/H^ CD302^-/-^] mice (Fig. 4C). In FNRG[EFKI TRIM26^H/H^ 3C] mice, we did observe a transient increase in viremia that resolved by day 42 (Fig. 4D). Upon examining the viremia of individual FNRG[EFKI TRIM26^H/H^ 3C] mice, we found that the majority (7/11) of the mice did not develop any viremia above baseline (Fig. 4E, G, I through K, N, and O). Two FNRG[EFKI TRIM26^H/H^ 3C] mice showed increased viremia at one time point post-infection (Fig. 4H and M), and one mouse showed consistent elevated viremia throughout infection (Fig. 4L). Taken together, these data suggest a more complex genetic background is necessary to improve upon the very limited permissiveness in FNRG[EFKI TRIM26^H/H^ 3C] mice.

### Humanization of the murine CypA locus fails to convey permissiveness in EFKI TRIM26^H/H^ CD302^-/-^ mice

**Fig 5:**
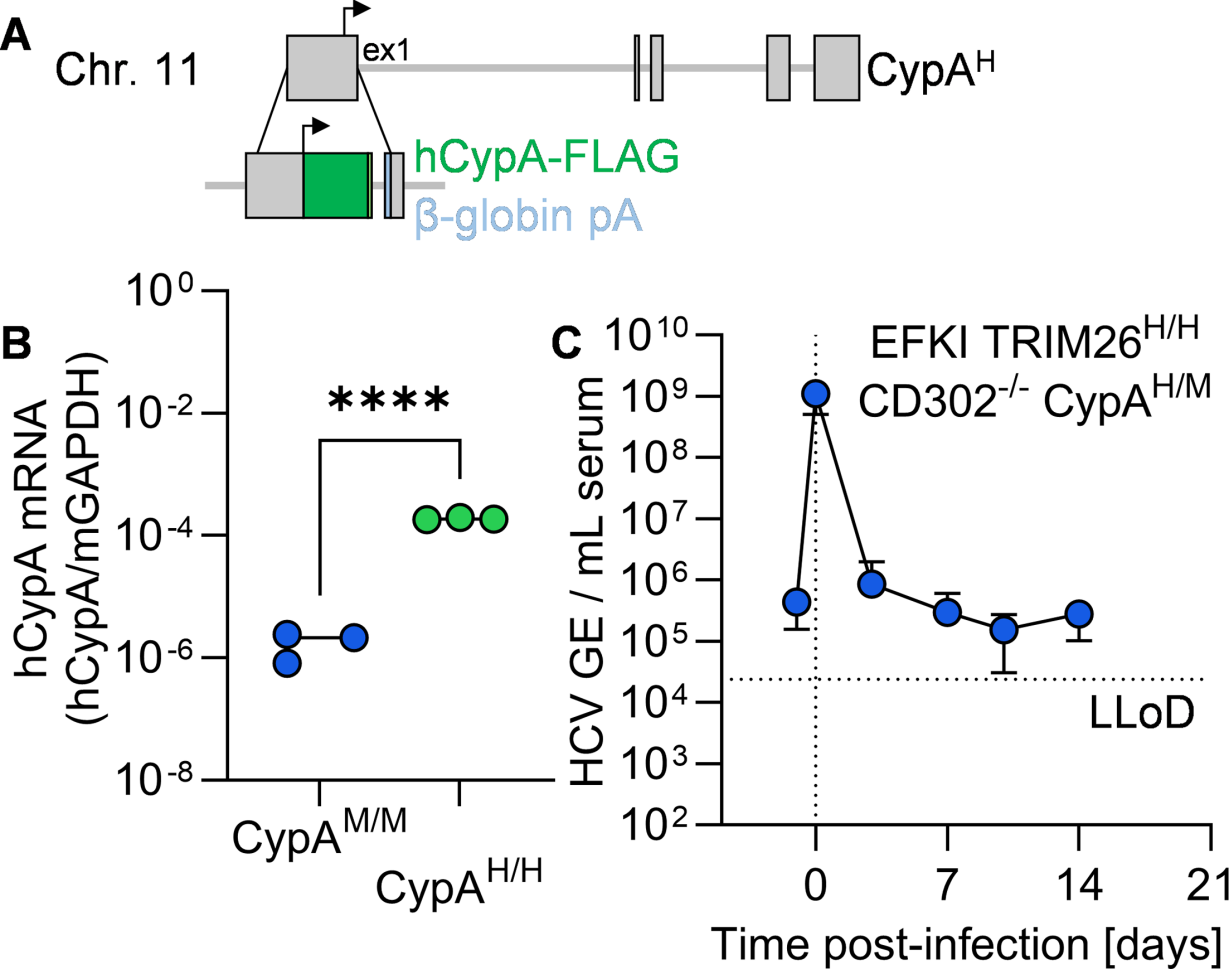
Humanization of CypA does not convey HCV permissiveness *in vivo*. (**A**) Schematic representation of the humanized CypA locus generated for this study. (**B**) Quantification of human CypA-specific transcript in liver tissue from EFKI and EFKI TRIM26^H/H^ CD302^-/-^ CypA^H/M^ mice. ****, *P* ≤ 0.0001. (**C**) Longitudinal HCV serum viremia in EFKI TRIM26^H/H^ CD302^-/-^ CypA^H/M^ mice (*N* = 8). LLoD, lower limit of detection.

The final HCV restriction factor that we humanized in this study was CypA, given the well-established insufficiency of the murine ortholog for HCV replication (35). In a similar fashion to TRIM26, we humanized the murine CypA locus by knocking in the coding sequence for human CypA immediately after the murine start codon (Fig. 5A). We included a C-terminal FLAG tag in this construct to aid in later detection, as hCypA has been previously shown to tolerate C-terminal epitope tags (35, 41). We detected significantly higher levels of human CypA RNA in liver tissue from CypA^H/H^ mice (Fig. 5B).

Given empirical difficulties in isolating CypA-homozygous pups in the EFKI TRIM26^H/H^ CD302^-/-^ background, we utilized heterozygotes for subsequent infections. Murine CypA does not exert a dominant negative effect when co-transduced with human CypA into CypA-deficient Huh7.5 cells (35), whereby we did not anticipate a significant difference between these mice and the ultimate EFKI TRIM26^H/H^ CD302^-/-^ CypA^H/H^ mice. Upon infection, we did not observe elevated HCV serum viremia above baseline in EFKI TRIM26^H/H^ CD302^-/-^ CypA^H/H^ mice (Fig. 5C).

### Variable HCV permissiveness of FNRG[EFKI TRIM26^H/H^ CD302^-/-^ CypA^H/M^] mice

**Fig 6:**
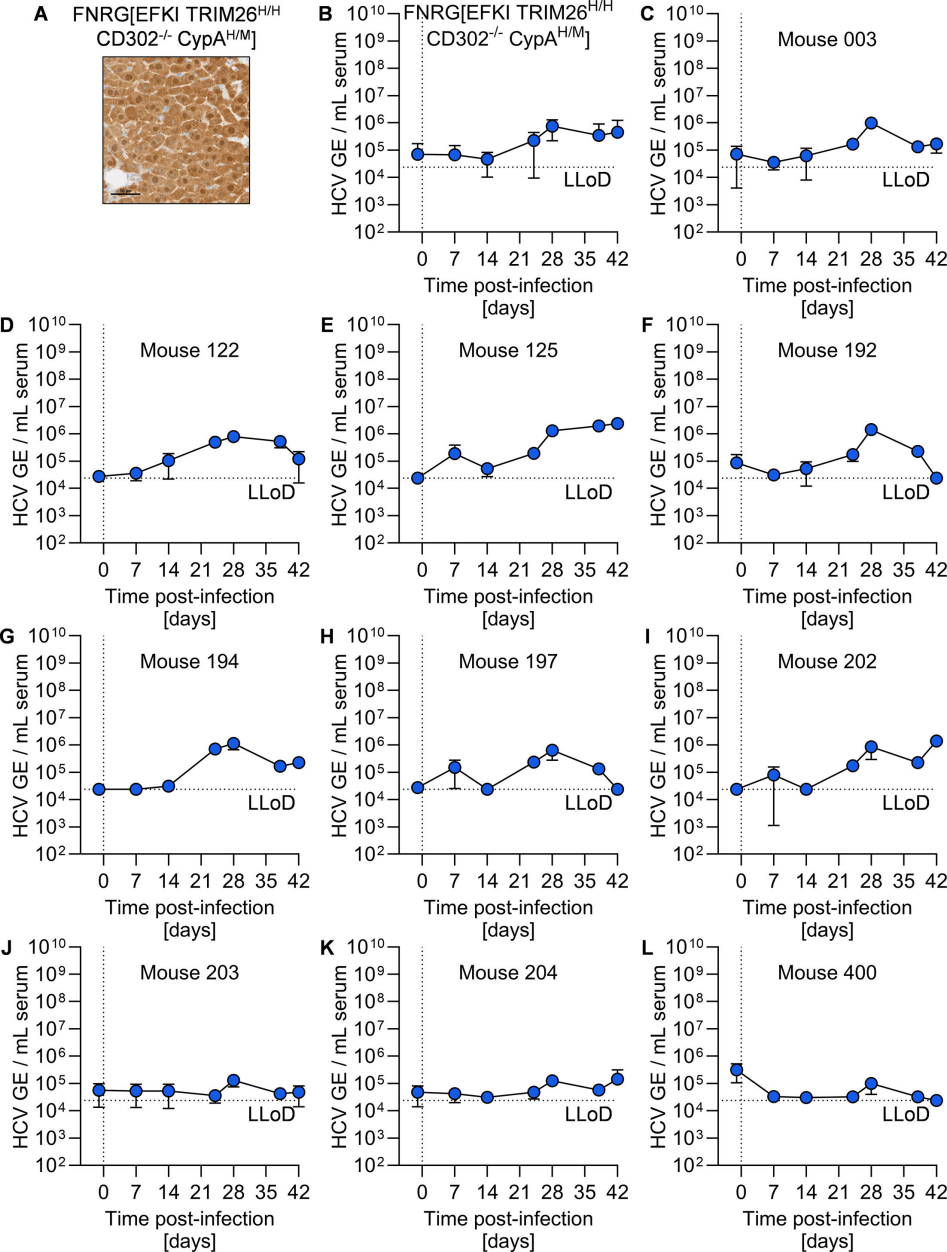
Immunodeficient mice harboring EFKI TRIM26^H/H^ CD302^-/-^ CypA^H/M^ hepatocytes are not permissive to HCV infection. (**A**) FAH staining (brown) of liver sections of a representative sample from an FNRG[EFKI TRIM26^H/H^ CD302^-/-^ CypA^H/M^] mouse. Scale bar = 50 µm. (**B**) Longitudinal HCV serum viremia in (**C**) FNRG[EFKI TRIM26^H/H^ CD302^-/-^ CypA^H/M^] mice (*N* = 10). LLoD, lower limit of detection. (**D–L**) Longitudinal serum viremia of individual mice in (**C**).

We finally generated and assessed HCV permissiveness in FNRG mice transplanted with EFKI TRIM26^H/H^ CD302^-/-^ CypA^H/M^ hepatocytes, the robust engraftment of which we confirmed by histology staining (Fig. 6A). The resultant cohort of FNRG[EFKI TRIM26^H/H^ CD302^-/-^ CypA^H/M^] mice did not develop viremia significantly above baseline (Fig. 6B). Upon examination of longitudinal viremia in the individual FNRG[EFKI TRIM26^H/H^ CD302^-/-^ CypA^H/M^] mice (Fig. 6C through L), we observed instances of transient viremia that resolved over time (Fig. 6C, D, and F through H), no development of viremia (Fig. 6J through L), and low-level, persistent viremia (Fig. 6E and I).

### Terminal liver HC viremia does not correlate with serum viremia

**Fig 7:**
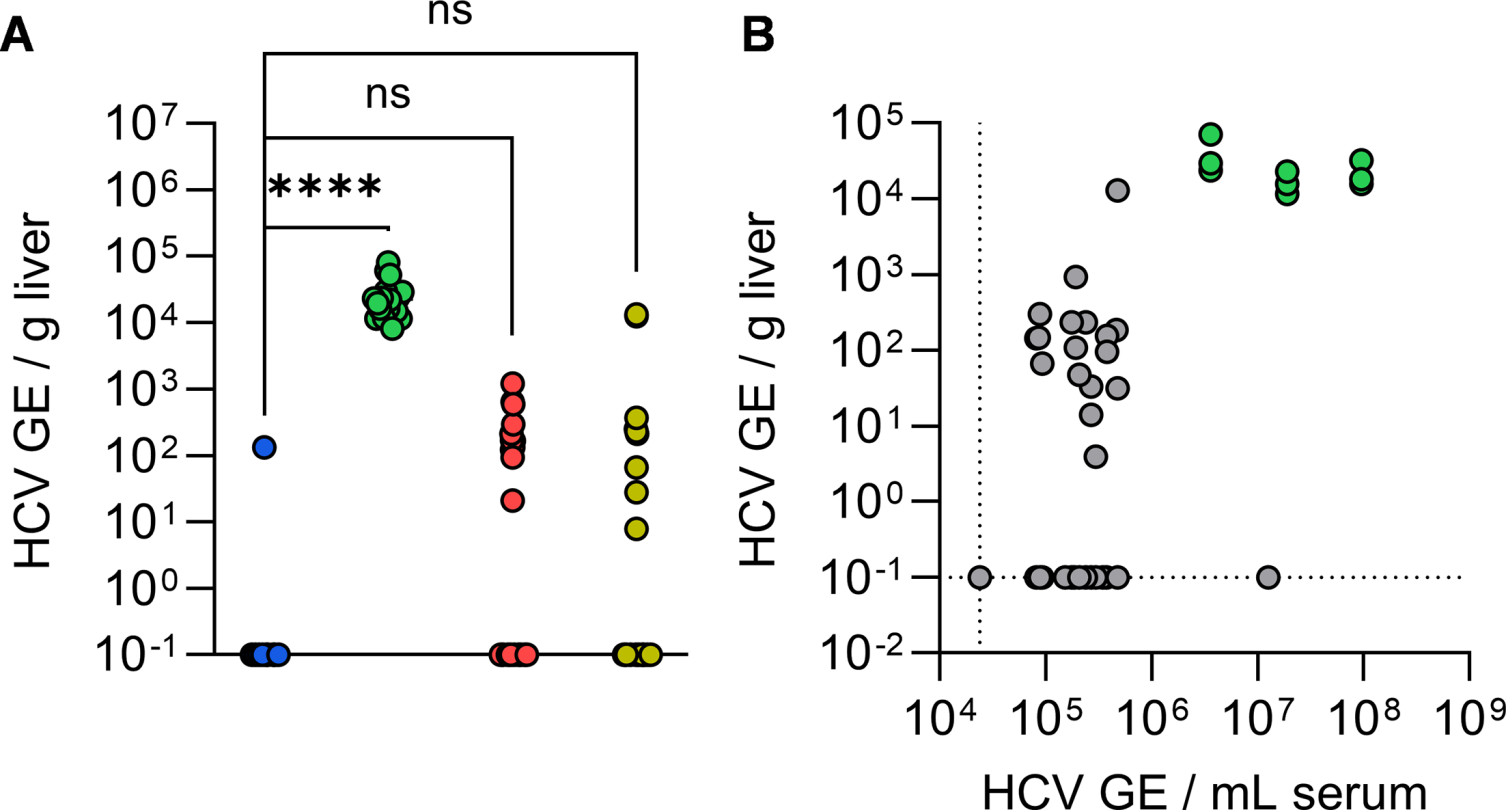
HCV-infected mice do not develop hepatic viral load. (**A**) Terminal hepatic viral load for C57BL/6 (blue) mice and FNRG mice transplanted with human (green), EFKI TRIM26^H/H^ CD302^-/-^ CypA^H/M^ (red), and EFKI TRIM26^H/H^ 3C (yellow) hepatocytes. ****, *P* ≤ 0.0001; ns, not significant. (**B**) Terminal hepatic viral load in (**A**) versus terminal serum viremia. Pearson’s *r* = 0.4224. Green, FNRG[Human] mice.

Building off of the positive serum viremia observed in several FNRG[EFKI TRIM26^H/H^ 3C] (Fig. 4) and FNRG[EFKI TRIM26^H/H^ CD302^-/-^ CypA^H/M^] (Fig. 6) mice, we aimed to detect HCV infection in livers from animals of these cohorts, in addition to C57BL/6 and FNRG[Human] samples as negative and positive controls, respectively (Fig. 7A). While human liver chimeric mice were highly viremic, we did not observe any significant increase in hepatic viral load cohort-wide among FNRG[EFKI TRIM26^H/H^ 3C] and FNRG[EFKI TRIM26^H/H^ CD302^-/-^ CypA^H/M^] mice (Fig. 7A) nor did hepatic viral load correlate with terminal serum viremia (Fig. 7B). These data suggest that, if HCV indeed replicated in hepatocytes of serum-positive animals, the levels are too low to exceed the limit of detection of our assay.

## DISCUSSION

Here, we present the generation and characterization of eight iteratively humanized mouse lines in pursuit of a model for HCV infection. We deliberately selected factors for which robust evidence has demonstrated their role in the inability of HCV to replicate in murine hepatocytes. These include murine genes incompatible with HCV biology (CD81, OCLN, TRIM26, CypA) (30, 32, 35) and those murine factors that actively inhibit infection (CD302, CR1L) (31). For each intermediate mouse line produced during the generation of EFKI TRIM26^H/H^ CD302^-/-^ CypAH/M and EFKI TRIM26^H/H^ 3C mice, we assessed susceptibility to HCV infection. For four of the most advanced of these lines, we successfully transplanted hepatocytes into immunodeficient FNRG mice and assessed the permissiveness of the resultant cohorts. Despite detecting a transient viremia in a subset of FNRG transplant recipients, we failed to generate a mouse line that permits robust HCV infection in 100% of mice.

The inherent necessity for any HCV mouse model is the modification of all host-viral interactions that prevent HCV from infecting wild-type mice. This requires an *a priori* understanding of all such factors, or at least those whose mutation is necessary to convey permissiveness. To this end, a more genetically complex mouse line will have to be generated on an EFKI TRIM26^H/H^ 3C CypA^H/H^ background. Other modifications in such a line may include humanizing factors central to HCV replication, such as phosphatidylinositol 4-kinase III alpha (42); knocking out interferon-stimulated genes (ISGs) for which the murine and human orthologs demonstrate divergent activity against HCV, such as receptor transporter protein 4 (RTP4) (43) (Ploss lab,M. P. Schwoerer, and A. Ploss, unpublished data); or overexpression of assembly factors that may encourage HCV release from the infected murine cell, such as apolipoprotein E (44).

The low-level permissiveness observed in a subset of FNRG[EFKI TRIM26^H/H^ 3C] (Fig. 4D through O) and FNRG[EFKI TRIM26^H/H^ CD302^-/-^ CypA^H/M^] (Fig. 6) mice is remarkable, given the lack of viremia detectable in their non-transplanted counterparts (Fig. 3E and 5C). This aligns with *in vivo* data indicating that blunting antiviral signaling can render HCV-susceptible mice permissive to low-level infection (45). This may indicate that the suite of ISGs generated in mice in response to HCV infection is more effective at extirpating viremia than their human orthologs, as has recently been shown in RTP4 (Ploss lab, M. P. Schwoerer, and A. Ploss, unpublished data), but may feasibly expand to other induced antiviral effectors. Upstream of ISGs, HCV may fail to antagonize antiviral signaling pathways in mouse cells to the same extent as in human cells. Whereas HCV NS3/4A cleaves both human and murine mitochondrial antiviral signaling protein (MAVS) (46, 47), it is not known whether other HCV antiviral mechanisms are conserved, such as the disruption of the interaction between stimulator of interferon (IFN) genes (STING) and TANK-binding kinase 1 (TBK1) by HCV NS4B (48), or HCV infection-driven degradation of signal transducer and activator of transcription 2 (STAT2) (49). Additionally, the very low-level replication in a minimally permissive mouse line may fail to antagonize IFN signaling at a level required to circumvent its antiviral effects. A necessary stipulation to these data is the high background observed in the quantitation of serum HCV GE levels, a necessary proxy for viremia given difficulties culturing serum-derived HCV. This stems both from the low volume of input serum (25 µL) and empirical background in this non-clinically standardized assay.

An overarching caveat to this work is the extent to which all infections herein have utilized a single HCV strain, the commonly used J6/JFH1-Jc1 intragenotypic (2a/2a) chimera of HCV (50). While this strain has proven indispensable for cell culture-based studies of HCV infection due to its high rates of replication, we note that this strain may not be ideal for *in vivo* applications. Indeed, there is limited correlation between *in vitro* and *in vivo* hepaciviral fitness, as has been established in HCV infections in chimpanzees (51) and NrHV infections in rodents (52). Beyond this, the extent to which JFH1 is derived from a case of fulminant HCV suggests that it may not be best suited for a model of chronic infection (53, 54). It will be necessary in future studies to broaden *in vivo* inocula to encompass a broader range of genotypes, particularly those that are not adapted to cell culture and those associated with higher chronicity rates, such as genotype 1b (55). We further note the extent to which we were unable to detect robust hepatic viral load in cohorts that presented with limited serum viremia (Fig. 7A), as well as the lack of correlation between these data and terminal serum viremia (Fig. 7B). We assert that this may reflect the low frequency of HCV+ hepatocytes present during *in vivo* infections; indeed, in liver samples from infected humans, detection and abundance of HCV antigens has long been known to bear limited correlation with serum viremia (56, 57), with often low frequencies of antigen-positive hepatocytes being detected (58–61). This further precludes attempts at leveraging immunofluorescence microscopy or histological methods to visualize HCV within liver sections in what may be very minimally permissive mouse lines.

Whereas our work has focused on modifying mice to render them permissive to HCV infection, it is logically consistent to adapt HCV to be infectious in mice. This has been utilized for a wide variety of positive-sense single-stranded (ss)RNA viruses, including ZIKV (26), SARS-CoV-1 (62) and −2 (63), coxsackievirus A6 (64), and dengue virus (65). These approaches take advantage of the error-prone nature of viral RNA-dependent RNA polymerases to generate a viral quasispecies that can overcome evolutionary bottlenecks and select for adaptive mutations. This complementary approach has succeeded in generating a strain of HCV that can utilize murine CD81 and OCLN for entry *in vitro* (66) and *in vivo* (67), albeit without replication in mice with blunted antiviral signaling.

More recently, a partially mouse-adapted strain of HCV (HCV Mad18) was developed by extensive passaging of HCV J6/JFH1-Jc1 in human and humanized murine hepatocytes (68). The resultant HCV Mad18 isolate was infectious in primary murine hepatocytes overexpressing human CD81 and OCLN and deficient in IFN-α/β receptor (IFNAR) (68). Given the extent to which mutations in the E1E2 glycoproteins are dispensable for Mad18 murine tropism *in vitro* (68), it is conceivable that HCV Mad18 has adapted to some combination of murine CypA or TRIM26 in order to promote infection. The consensus sequence of the HCV Mad18 quasispecies contains 10 nonsynonymous mutations in NS5A and two in NS5B (68). Whether these mutations facilitate HCV Mad18 NS5A binding to murine CypA or Mad18 NS5B to murine TRIM26 remains unexplored. Indeed, it may be the case that EFKI TRIM26^H/H^ 3C CypA^H/H^ mice with genetically modified IFN signaling may be permissive to HCV Mad18. Such a capability would open the door to studies into *bona fide* long-term HCV infection in an immunocompetent mouse line derived from this work.

## MATERIALS AND METHODS

### Generation of HCV RNA and viral stocks

HCV viral RNA was produced via *in vitro* transcription of an XbaI-linearized J6/JFH1 (Jc1)(50) plasmid using the HiScribe T7 High Yield RNA Synthesis Kit (New England Biolabs, Ipswich, MA, USA) as outlined in the user manual. Viral RNA was purified using the MEGAclear Transcription Clean-Up Kit (Thermo Fisher Scientific, Waltham, MA, USA) following manufacturer’s instructions, and quality control was performed by gel electrophoresis to ensure no significant RNA degradation. Viral RNA stocks were stored as 5 µg aliquots at −80°C. RNA was electroporated into Huh7.5-1 cells (69). The pellet was resuspended in the appropriate volume of ice-cold Dulbecco's phosphate buffered saline (DPBS) to achieve a concentration of 1.5E7 cells/mL. 6E6 cells were then electroporated in a 2 mm path length electroporation cuvette (BTX Harvard Apparatus, Holliston, MA, USA) with 5 µg of viral RNA using an ECM 830 Square Wave Electroporation System (BTX) at the following settings: five pulses, 99 µs per pulse, 1.1 s pulse intervals, 860V. Following a 10-min incubation at room temperature (RT), the electroporated cells were seeded into 150 mm plates and maintained in Dulbecco's modified Eagle's medium (DMEM) with 5% (vol/vol) fetal bovine serum (FBS) (Bio-Techne, Minneapolis, MN, USA) and 1× non-essential amino acids (Gibco, Waltham, MA, USA). Media was changed 1 day post-electroporation, and supernatants were collected twice daily from days 4 through 6 and stored at 4°C. The pooled supernatants were passed through a 0.22 µm vacuum filter and subsequently concentrated to ~40 mL in 100 kDa molecular weight cutoff (MWCO) Amicon Ultra-15 Centrifugal Filter Units (Millipore Sigma, Allentown, PA, USA).

### Quantification of HCV titer by limiting dilution

The TCID50/mL of concentrated virus was determined after one freeze-thaw by limiting dilution assay. Huh7.5 cells were seeded in a 96-well plate at a density of 6,400 cells/well. Fifty microliters of 10-fold serial dilutions (from 1:1E2 to 1:1E7) of virus was added to each column of wells, with eight wells receiving each dilution. After removal of the inoculum 6–8 h post-infection, cells were washed with unsupplemented DMEM and cultured in 200 µL DMEM containing 10% (vol/vol) FBS and 1% (vol/vol) penicillin/streptomycin. On day 3 post-infection, cells were fixed and permeabilized in ice-cold 100% methanol for 30 min at −20°C. Cells were blocked in 1× phosphate buffered saline (PBS) containing 0.1% (vol/vol) Tween-20, 1% (wt/vol) bovine serum albumin (BSA), and 0.2% (wt/vol) skim milk for 30 min at RT. Cells were then treated with PBS containing 3% H_2_O_2_ for 5 min at RT. Cells were then stained with a mouse anti-HCV NS5A monoclonal antibody (clone 9E10 [70], 220 ng/mL, 50 µL/well) for 1 h at RT, followed by a horseradish peroxidase (HRP)-conjugated goat anti-mouse polyclonal antibody (Invitrogen, Waltham, MA, USA, 5 µg/mL, 50 µL/well). HRP signal was detected using 3,3-diaminobenzidine (DAB) Peroxidase (HRP) Substrate Kit (Vector Laboratories, Newark, CA, USA). TCID50/mL was calculated using the Reed and Muench method (71).

### *In vivo* experiments

All mice were bred and generated in the Laboratory Animal Resource Center of Princeton University. C3-deficient mice (38) were commercially obtained from the Jackson Laboratory (strain #:029661). EFKI mice were generated as described previously (36).

### 2C-HR-CRISPR reagents

Cas9 mRNA was synthesized as previously described (39, 72). Briefly, pCS2-Cas9 plasmid (Addgene 122948) was linearized with NotI restriction digestion (New England Biolabs, Ipswich, MA, R3189L) and used as a template for *in vitro* transcription using a mMESSAGE mMACHINE SP6 Transcription Kit (Thermo Fisher Scientific, Waltham, MA, AM1340). Cas9 mRNA was purified with the RNeasy Mini Kit (Qiagen, Germantown, MD, 74104) using the cleanup protocol according to manufacturer’s instructions.

Targeting vectors for human TRIM26 and human CypA-FLAG were cloned by PCR amplifying ~1 kb homology arms (5′ and 3′) from genomic DNA, immediately up- and downstream of the sgRNA cut site. The human TRIM26 and hCypA-FLAG knock-in sequences were derived from the human ORFeome library v.8.1 (73). Final targeting plasmids were prepared using an endotoxin-free Maxi prep kit.

sgRNA sequences (Table 1) were designed using CRISPOR design tool (http://crispr.mit.edu/). sgRNA targets were selected to delete exons upon recombination for cut sites (CR1L and CD302 knockout). The TRIM26^H/H^ and CypA^H/H^ knock-in lines were created by targeting cut sites with close proximity to the endogenous start codon and using 2C-HR-CRISPR to create the knock-in allele as previously described (72).

**TABLE 1:** Guide RNAs utilized to generate transgenic mouse lines

Guide	Sequence (5′–3′)
Trim26-35rev	CUGCCAUUGCACCCUUAGGU
Trim26-52	CCCCCAACCUAAGGGUGCAA
CypA-6rev	CGACAGUGGCGUCUGCAAAG
mCD302-Ex1a	GAGCGAGGACAGCGCUGCGU
mCD302-Ex6a	GAUCGCUAGCACAGUAACUC
mCr1l-Ex1a	CUUGACUCCUCCCCGGCCGA
mCr1l-Ex10a	GAGUGAGUGAAUUCCGUGCU

### Editing embryos

Mice were generated via two-cell cytoplasmic injection as described previously (72). TRIM26^H/H^ mice were generated in EFKI mice on a C57BL/6 background using guides Trim26-35rev and Trim26-52 and CypA^H/M^ mice using guide CypA-6rev.

Female mice aged between 4 and 8 weeks of age were each injected with 7.5 IU pregnant mare serum gonadotropin (Sigma-Aldrich, St. Louis, MO, G4527) and 7.5 IU human chorionic gonadotropin (hCG, Sigma-Aldrich, St. Louis, MO, 9002-61-3), 47 h apart. The females were then mated with males over 8 weeks of age. Vaginal plugs were checked the following morning. Plugs were counted as embryonic day (E) 0.5. Two-cell embryos were collected from superovulated females on E1.5 in M2 media (Cytospring, Mountain View, CA M2116).

Microinjection mixes for human TRIM26 and CypA-FLAG knock-in were prepared in 15 µL total nuclease-free injection buffer (10 mM Tris-HCl, pH 7.4, and 0.25 mM EDTA) with 30 ng/µL targeting plasmid, 100 ng/µL Cas9 mRNA, and 50 ng/µL sgRNA. Microinjection mix for the CD302 deletion was adapted from the methods outlined above to include two sgRNA guides with no repair template to induce a deletion of the DNA between the two guides via non-homologous end joining (NHEJ) of the cut sites. Microinjection mix was prepared in 15 µL total nuclease-free injection buffer (10 mM Tris-HCl, pH 7.4, and 0.25 mM EDTA) with 100 ng/µL Cas9 mRNA and 37.5 ng/µL of each sgRNA. Microinjection mix for the CR1L deletion was prepared in 15 µL total nuclease-free injection buffer (10 mM Tris-HCl, pH 7.4, and 0.25 mM EDTA) with 10 ng/µL Cas9 mRNA and 3.75 ng/µL of each sgRNA.

Microinjection was performed under negative capacitance using a Leica microscope and micromanipulators (Leica Microsystems, Wetzlar, Germany). Injection pressure was provided by a FemtoJet (Eppendorf, Hamburg, Germany) and negative capacitance was generated with a Cyto721 intracellular amplifier (World Precision Instruments, Sarasota, FL). Microinjections were performed in M2 media (Cytospring, Mountain View, CA) in an open glass chamber. After microinjections, embryos were immediately transferred into pseudopregnant CD-1 IGS females (Charles River strain 022) and gestated until birth. Founder mice were identified using genotyping PCR. Founder mice were outcrossed to generate N1 mice, and N1 animals were sequence-verified.

### Genotyping

To test for CD81^EL2[H/H]^ and OCLN^EL2[H/H]^, we conducted qPCR on ear clippings as described previously (36) (Table 2). To test for C3^-/-^, CD302^-/-^, CR1L^-/-^, and TRIM26^H/H^, mouse genotypes from ear clips were determined using real-time PCR with specific probes designed for each gene (Transnetyx, Cordova, TN); Transnetyx probe sequences are proprietary. To test for CypA^H/H^, we conducted two diagnostic PCR reactions to screen for the novel junction present in knockout mice (Table 2). The following PCR conditions were used for PCR BD: 1 cycle of 95°C (120 s); 35 cycles of 95°C (20 s), 63°C (30 s), 72°C (14 s); 1 cycle of 72°C (120 s). The following PCR conditions were used for PCR B.2: 1 cycle of 95°C (120 s); 35 cycles of 95°C (20 s), 56°C (30 s), 72°C (13 s); 1 cycle of 72°C (120 s). In mice harboring the CypA^M^ allele, PCR BD will produce a band of 204 bp; in mice harboring the CypA^H^ allele, PCR B.2 will produce a band of 198 bp.

**TABLE 2:** Primers used for genotyping transgenic mouse lines

Primer	Purpose	Sequence (5′–3′)
PU-O-3812	mCD81^hEL2^ qPCR primer 1	CCAAGGCTGTGGTGAAGACTTTC
PU-O-3814	mCD81^hEL2^ qPCR primer 2	GGCTGTTCCTCAGTATGGTGGTAG
PU-O-3812	mCD81^WT^ qPCR primer 1	CCAAGGCTGTGGTGAAGACTTTC
PU-O-3813	mCD81^WT^ qPCR primer 1	TGTTCTTGAGCACTGAGGTGGTC
PU-O-1235	mOCLN^hEL2^ qPCR primer 1	GTGTTTATTGCCACGATCGTGT
PU-O-1236	mOCLN^hEL2^ qPCR primer 2	AAATTGGTTGCAGAGGGCATAT
PU-O-1237	mOCLN^WT^ qPCR primer 1	CTCTTTGGAGGAAGCCTAAACTACC
PU-O-1238	mOCLN^WT^ qPCR primer 1	AAACTGGTTGCAGATCATATAT
PU-O-1000	mGAPDH qPCR primer 1	ACGGCCGCATCTTCTTGTGCA
PU-O-1001	mGAPDH qPCR primer 2	ACGGCCAAATCCGTTCACACC
PU-O-12428	CypA PCR BD primer 1	GTGATTGATCCAGGTCCGGG
PU-O-12430	CypA PCR BD primer 2	ACCCGACCTCGAAGGAGAC
PU-O-11822	CypA PCR B.2 primer 1	CATCTGCACTGCCAAGACTGAG
PU-O-11765	CypA PCR B.2 primer 2	GAGTTATCTCACTTATCGTCGTCATCC

### RNA extraction from mouse liver tissue and gene expression analysis by RT-qPCR

Liver tissue was divided into three portions and stored at either −80°C in RNALater, −80°C embedded in optimal cutting temperature (OCT) solution, or 4°C in neutral-buffered formalin (NBF) (Sigma-Aldrich, St. Louis, MO, USA). NBF samples were utilized for histological analyses. Stainless steel beads (5 mm, Qiagen, Hilden, Germany) and 350 µL lysis buffer were added to sample tubes containing 10 mg–50 mg of RNALater-preserved liver tissue and homogenized using a TissueLyser LT (Qiagen, Hilden, Germany). Total RNA was isolated from lysate using the KingFisher Flex System (Thermo Fisher Scientific, Waltham, MA, USA). For gene expression analysis, liver RNA was diluted 1:100 in H_2_O and analyzed by RT-qPCR using gene-specific primers (Table 3).

**TABLE 3:** Primers used for phenotyping transgenic mouse lines

Primer	Purpose	Sequence (5′–3′)
PU-O-1235	mOCLN^hEL2^ qPCR primer 1	GTGTTTATTGCCACGATCGTGT
PU-O-1236	mOCLN^hEL2^ qPCR primer 2	AAATTGGTTGCAGAGGGCATAT
PU-O-1237	mOCLN^WT^ qPCR primer 1	CTCTTTGGAGGAAGCCTAAACTACC
PU-O-1238	mOCLN^WT^ qPCR primer 1	AAACTGGTTGCAGATCATATAT
PU-O-1000	mGAPDH qPCR primer 1	ACGGCCGCATCTTCTTGTGCA
PU-O-1001	mGAPDH qPCR primer 2	ACGGCCAAATCCGTTCACACC
PU-O-11822	hCypA qPCR primer 1	CATCTGCACTGCCAAGACTGAG
PU-O-11765	hCypA qPCR primer 2	GAGTTATCTCACTTATCGTCGTCATCC
PU-O-11930	hTRIM26 qPCR primer 1	TGCACTACTACTGTGAGGACG
PU-O-11931	hTRIM26 qPCR primer 2	TCCTTAGGGTACTCAGGTGGT
PU-O-4815	mCD302 qPCR primer 1	CAAGATGGTGAGGACCTAG
PU-O-4816	mCD302 qPCR primer 2	AATACTTCTTGTCATATGGGATTGC
PU-O-12708	mCR1L qPCR primer 1	TTCTTGCTGCCATTTACTTTGGG
PU-O-12709	mCR1L qPCR primer 2	CGTCCAGGTTGAGTCTTGTTTG

### C3 complement ELISA

To quantify C3 concentrations, serum was analyzed using a Mouse Complement C3 ELISA Kit (MyBioSource, Inc, San Diego, CA, USA) following manufacturer’s instructions.

### Mouse hepatocyte isolation and transplantation

Murine hepatocyte isolation and transplantation into FNRG mice were conducted as described previously (74). In brief, mice were anesthetized by intraperitoneal injection of a mixture of 100 mg/kg ketamine and 10 mg/kg xylazine. Livers were perfused through the portal vein with a chelating solution (0.01M HEPES, pH 7.3, and 0.5 mM EGTA pH 8.0 in Ca^2+^/Mg^2+^-free Earle's balanced salt solution[EBSS]) at a flow rate of 2 mL/min until the liver blanched, followed by 40 mL collagenase solution (0.01M HEPES pH 7.3 and 1 mg/mL collagenase type II in EBSS with Ca^2+^, Mg^2+^, and phenol red). The digested liver was cut into pieces, transferred into a washing solution (0.01M HEPES, pH7.3, and 10% FBS in DMEM), passed through a 100 µm cell strainer, washed, and passed through a 100 µm cell strainer. The resulting cell suspension was passed through a 70 µm cell strainer. The cell suspension was washed three more times with spinning steps at 140 g for 5 min to remove unwanted cellular debris. Cells were resuspended in HyClone DMEM (Cytiva, Marlborough, MA, USA), and cell viability was assessed through trypan blue exclusion.

### Engraftment of mouse hepatocytes into FNRG recipients

The generation of FAH^-/-^ NOD.Cg-Rag1^tm1Mom^IL2rg^tmlWjl^/SzJ IL2Rg^null^ (FNRG) mice has been previously described (11). FNRG mice maintained on water supplemented with 10% (wt/vol) NTBC (Yecuris Inc., Tualatin, OR, USA) to block the build-up of metabolites to toxic concentrations. To facilitate hepatic engraftment, female FNRG mice older than 7 weeks of age were injected intrasplenically with ca. 1.0E6 hepatocytes freshly isolated from donor strains as specified in the results section. Following transplantation, FNRG mice were given water lacking NTBC for 9 days, followed by 7 days with 10% NTBC, 7 days with water lacking NTBC, and then 4 days with 10% NTBC. Following this, mice were solely provided water without NTBC.

### HCV RNA isolation from serum

Mouse blood was collected via submandibular bleeding at the aforementioned intervals. Serum was separated from cells by centrifuging the coagulated blood (3,500 rpm, 10 min, room temperature) and collecting the supernatant. Total RNA was isolated from 25 µL serum using the Zymo Viral RNA extraction kit (Genesee Scientific, El Cajon, CA, USA) or the KingFisher Flex System (Thermo Fisher Scientific, Waltham, MA, USA), and the HCV genome copy number was quantified by one-step RT-qPCR using a Multicode-RTx HCV RNA kit (Luminex Corporation, Austin, TX, USA) and a StepOne Real-Time PCR (Applied Biosystems, Waltham, MA, USA), according to manufacturer’s instructions.

### HCV RNA isolation from liver tissue

Liver tissue was stored at −80°C in RNALater. Stainless steel beads (5 mm, Qiagen, Hilden, Germany) and 350 µL lysis buffer were added to sample tubes containing 10 mg–50 mg liver tissue and homogenized using a TissueLyser LT (Qiagen, Hilden, Germany). Total RNA was isolated from lysate using the Monarch Total RNA Miniprep Kit (New England Biolabs, Ipswich, MA, USA) or the KingFisher Flex System (Thermo Fisher Scientific, Waltham, MA, USA), and the HCV genome copy number was quantified by one-step RT-qPCR using a Luna Probe One-Step RT-qPCR 4× Mix with UDG (New England Biolabs, Ipswich, MA, USA) and a StepOne Real-Time PCR (Applied Biosystems, Waltham, MA, USA). The primer and probe sequences are listed in Table 4. For RT-qPCR, the following cycling conditions were used: 1 cycle of 63°C for 3 min; 1 cycle of 95°C for 30 s; 45 cycles of 95°C for 15 s, 60°C for 30 s; 1 cycle of 40°C for 30 s.

**TABLE 4:** Primers used for quantification of HC viremia in liver samples

Primer	Purpose	Sequence (5′–3′)
PU-O-13475	HCV RT-qPCR FP	TCTGCGGAACCGGTGAGTA
PU-O-13476	HCV RT-qPCR RP	GGGCATAGAGTGGGTTTATCCA
PU-O-13477	HCV RT-qPCR Probe	[6FAM]-AAAGGACCCAGTCTTCCCGGCAA-[TMR]

### Histology processing, chromogenic immunohistochemistry, and whole slide scanning

Tissue samples were fixed for a minimum of 72 h in 4% (wt/vol) paraformaldehyde and dehydrated for a minimum of 72 h in 70% (vol/vol) ethanol before processing in a Tissue-Tek VIP-5 automated vacuum infiltration processor (Sakura Finetek USA, Torrance, CA, USA) and embedded in paraffin using a HistoCore Arcadia paraffin embedding machine (Leica, Wetzlar, Germany). A total of 5 µm tissue sections were generated using a RM2255 rotary microtome (Leica, Wetzlar, Germany) and transferred to positively charged slides. A Ventana Discovery Ultra tissue autostainer (Roche Diagnostics, Indianapolis, IN, USA) was used for chromogenic immunohistochemistry. For FAH, an FAH rabbit primary polyclonal antibody was diluted to 1:100 in Ventana antibody diluent with casein (Roche) and incubated with tissue samples at RT for 3 h (Invitrogen: PA5-42049), followed by incubation with a secondary goat anti-rabbit HRP-polymer antibody (Vector Laboratories, Burlingame, CA, USA) for 20 min at 37°C, and developed with DAB and hematoxylin counterstain (Roche). For CD81 phenotyping, the same procedure was conducted using rabbit anti-CD81 clone D3N2D (Cell Signaling Technology, Danvers, MA, USA) at a dilution of 1:100 and incubation of 24 h. Histology images were acquired using a PhenoImager whole slide scanner (Akoya Biosciences, Marlborough, MA, USA) for figure preparation.

## Data Availability

All data supporting the conclusions of this study are reported in the paper. The raw data are available from the corresponding author with no restrictions upon reasonable request. Source data are provided with this paper. All primary data are available via Princeton Data Commons: https://doi.org/10.34770/zp7h-3b04.
